# Expanding the therapeutic role of highly purified cannabidiol in monogenic epilepsies: A multicenter real‐world study

**DOI:** 10.1111/epi.18378

**Published:** 2025-03-24

**Authors:** Emanuele Cerulli Irelli, Adolfo Mazzeo, Roberto H. Caraballo, Marco Perulli, Patrick B. Moloney, Javier Peña‐Ceballos, Marica Rubino, Katarzyna M. Mieszczanek, Andrea Santangelo, Laura Licchetta, Valentina De Giorgis, Gabriela Reyes Valenzuela, Susanna Casellato, Elisabetta Cesaroni, Francesca F. Operto, Jana Domínguez‐Carral, Alia Ramírez‐Camacho, Alessandro Ferretti, Giuseppe Santangelo, Angel Aledo‐Serrano, Andrea Rüegger, Maria M. Mancardi, Giulia Prato, Antonella Riva, Luca Bergonzini, Duccio M. Cordelli, Paolo Bonanni, Francesca Bisulli, Giancarlo Di Gennaro, Sara Matricardi, Pasquale Striano, Norman Delanty, Carla Marini, Domenica Battaglia, Carlo Di Bonaventura, Georgia Ramantani, Elena Gardella, GENE‐CBD Study Group, Alessandra Morano, Nicola Simeone, Claudia Cuccurullo, Massimo Scacciati, Alice Bonuccelli, Maddalena Bianchetti, Alberto Verrotti, Marta Piccioli, Massimiliano Celario, Elena Tartara, Giulia Balletto, Chiara Bagliani, Beatriz Gonzalez Giraldez, Pasquale Parisi, Carmen Salluce, Emanuele Bartolini, Maria Del Carmen Fons‐Estupiña, Federico Ramos, Alberto Danieli, Alessandra Rossi, Elisabetta Pantaloni, Ana Laura Fernandez‐Perrone, Giorgio Magli, Ilaria Onida, Giulia Bruschi, Lorenzo Muccioli, Tomasz S. Mieszczanek, Trine Hammer, Francesca Furia, Alessandro Orsini, Antonietta Coppola

**Affiliations:** ^1^ Department of Human Neurosciences Sapienza University Rome Italy; ^2^ Department of Neurology Hospital de Pediatría “Prof. Dr. Juan P. Garrahan” Buenos Aires Argentina; ^3^ Pediatric Neurology and Psychiatric Unit Fondazione Policlinico Universitario Agostino Gemelli IRCCS Rome Italy; ^4^ Department of Neurology Mater Misericordiae University Hospital Dublin Ireland; ^5^ Department of Neurology Beaumont Hospital Dublin Ireland; ^6^ Department of Neuroscience, Reproductive Sciences, and Odontostomatology Federico II University of Naples Naples Italy; ^7^ Danish Epilepsy Center Dianalund Denmark; ^8^ Department of Clinical and Experimental Medicine University of Pisa Pisa Italy; ^9^ Istituto di Ricovero e Cura a Carattere Scientifico (IRCCS), Istituto Delle Scienze Neurologiche di Bologna Bologna Italy; ^10^ Brain and Behavioral Sciences Department University of Pavia Pavia Italy; ^11^ Department of Child Neurology and Psychiatry, Childhood and Adolescence Epilepsy Center Istituto di Ricovero e Cura a Carattere Scientifico (IRCCS), Mondino Foundation Pavia Italy; ^12^ Child Neuropsychiatry Unit, Department of Woman's and Child's Health, Center of Pediatric Epilepsies, Azienda Ospedaliera Universitaria, Sassari University of Sassari Sassari Italy; ^13^ Child Neurology and Psychiatric Unit, Pediatric Hospital G. Salesi Azienda Ospedaliero‐Universitaria Delle Marche Ancona Italy; ^14^ Department of Science of Health, School of Medicine University of Catanzaro Catanzaro Italy; ^15^ Epilepsy Unit, Department of Child Neurology Institut de Recerca Sant Joan de Déu Barcelona Spain; ^16^ Department of Neuroscience, Mental Health, and Sense Organs, Faculty of Medicine and Psychology Sant'Andrea Hospital Rome Italy; ^17^ Child Neuropsychiatry Department Itituto Mediterraneo di Eccellenza Pediatrica‐Azienda di Rilievo Nazionale ad Alta Sspecializzazione, Civico Palermo Italy; ^18^ Epilepsy and Neurogenetics Unit, Vithas La Milagrosa University Hospital Vithas Hospital Group Madrid Spain; ^19^ Department of Neuropediatrics University Children's Hospital Zurich Switzerland; ^20^ Child Neuropsychiatry Unit, member of the European Reference Network for Rare and Complex Epilepsies IRCCS Istituto Giannina Gaslini Genoa Italy; ^21^ Department of Neurosciences, Rehabilitation, Ophthalmology, Genetics, Maternal and Child Health University of Genoa Genoa Italy; ^22^ Epilepsy and Clinical Neurophysiology Unit Scientific Institute for Research, Hospitalization and Healthcare, E. Medea Conegliano Italy; ^23^ Department of Biomedical and Neuromotor Sciences University of Bologna Bologna Italy; ^24^ Istituto di Ricovero e Cura a Carattere Scientifico “Neuromed” Pozzilli Italy; ^25^ Department of Pediatrics University of Chieti‐Pescara Chieti Italy; ^26^ School of Pharmacy and Biomolecular Sciences Royal College of Surgeons in Ireland Dublin Ireland; ^27^ Research Ireland FutureNeuro Centre Dublin Ireland; ^28^ University of Southern Denmark Odense Denmark

**Keywords:** CBD, developmental and epileptic encephalopathy, effectiveness, epilepsy, intellectual disability, Lennox–Gastaut syndrome

## Abstract

**Objective:**

This real‐world, retrospective, multicenter study aims to investigate the effectiveness of highly purified cannabidiol (CBD) in a large cohort of patients with epilepsy of genetic etiology due to an identified monogenic cause. Additionally, we examine the potential relationship between specific genetic subgroups and treatment response.

**Methods:**

This study was conducted across 27 epilepsy centers and included patients with monogenic epileptic disorders (pathogenic or likely pathogenic variants) who were treated with highly purified CBD for at least 3 months.

**Results:**

A total of 266 patients (135 females, 50.8%) with monogenic epilepsies were included with a median age at CBD initiation of 12 years (interquartile range [IQR] = 7–19) and a median follow‐up duration of 17 months (IQR = 12–24). Overall, 77 different monogenic epilepsies have been included, with the most common genes being *SCN1A* (32.3%), *TSC2* (13.5%), *CDKL5*, and *MECP2* (4.5% each). The mean seizure reduction at the last follow‐up was 38.6%, with 47.5% of patients achieving ≥50% seizure reduction and 7.4% achieving seizure freedom. The Clinical Global Impression scale indicated improvement in 65.8% of patients. The general linear mixed model revealed that a shorter maximum duration of seizure freedom before CBD initiation and a higher degree of intellectual disability were independently associated with lower CBD effectiveness. Conversely, no significant differences in seizure outcome were observed across different epilepsy syndromes (Lennox–Gastaut syndrome, Dravet syndrome, tuberous sclerosis complex epilepsy, and other developmental and epileptic encephalopathy), between approved indications and off‐label use, or between concomitant clobazam use or not.

**Significance:**

This study supports CBD as a potential treatment for monogenic epilepsies beyond its licensed indications, demonstrating comparable effectiveness between approved and off‐label use and suggesting genetic subgroups with promising treatment responses.

**Key points**

- In a cohort of difficult‐to‐treat monogenic epilepsies, CBD reduced seizures by 38.6%, with 47.5% of patients achieving ≥50% reduction.
- Effectiveness was comparable between approved and off‐label CBD use.
- No differences in seizure outcome were observed across different syndromes, including LGS, TSC‐epilepsy, DS, and other DEEs.
- Shorter prior seizure freedom duration and greater intellectual disability predicted lower CBD response.
- This study's findings support CBD as a treatment option for diverse monogenic epilepsies beyond previously approved indications.

## 1 INTRODUCTION

The management of epilepsy has evolved significantly over the past few decades, with a growing emphasis on personalized epilepsy management tailored to individual patient profiles.^1^, ^2^ Cannabidiol (CBD), a nonpsychoactive cannabinoid derived from the *Cannabis sativa* plant, has gained attention in recent years for its potential therapeutic effects in the context of drug‐resistant epilepsy.^3^ Based on the results of different randomized controlled trials (RCTs), the US Food and Drug Administration and the European Medicine Agency approved highly purified CBD for the treatment of drug‐resistant seizures in two distinct developmental and epileptic encephalopathies (DEEs), namely, Dravet syndrome (DS) and Lennox–Gastaut syndrome (LGS).^4^, ^5^ Additionally, highly purified CBD has been approved for the treatment of tuberous sclerosis complex (TSC)‐related epilepsy, including the full spectrum of TSC‐associated refractory epilepsy, from DEEs to focal epilepsy.^6^

Despite the well‐documented efficacy reported in these syndromes, the use of CBD as a treatment for a broader range of monogenic epilepsies remains an area of active investigation. To date, few studies, particularly small case series, have explored the effectiveness of highly purified CBD in monogenic epilepsies other than TSC and DS caused by *SCN1A* pathogenic variants. These include epilepsies caused by pathogenic variants of *CDKL5*, *SYNGAP1*, *MECP2*, and *KCNT1*.^7^, ^8^, ^9^, ^10^, ^11^ Monogenic epilepsies, caused by single‐gene pathogenic variants, are distinct from other epilepsy etiologies due to specific molecular mechanisms that can promote epileptogenesis, including effects on cell migration, neuronal excitability, and synaptic function.^12^ Detailed information regarding the effectiveness of CBD within this etiological group and according to particular genetic variants can guide personalized treatment strategies and improve patient outcomes. A recent study by Nissenkorn and colleagues investigated the differential effectiveness of perampanel across various monogenic epilepsies. This study reported a high effectiveness of this antiseizure medication (ASM) in patients with monogenic epilepsies, particularly in certain single‐gene etiologies, underscoring the importance of tailoring treatments to specific epilepsy etiologies.^13^

This real‐world retrospective multicenter study aims to evaluate the effectiveness and safety of highly purified CBD in a large cohort of patients with epilepsy of genetic etiology due to an identified monogenic cause. Additionally, we aim to investigate the potential relationship between specific genetic variants and CBD response.

## 2 MATERIALS AND METHODS

### 2.1 Study participants and clinical data collection

This was a multicenter retrospective study conducted across 27 international specialized epilepsy centers for both adults and children, most of which are members of the European Reference Network for Rare and Complex Epilepsies (EpiCARE). The study was approved by the institutional/regional ethical committee (approval number 7671, 0534/2024), and informed consent was obtained from all participants. The study was conducted according to the STROBE (Strengthening the Reporting of Observational Studies in Epidemiology) guidelines.

Patients attending participating centers who were prescribed >99% highly purified CBD (Epidiolex, Jazz Pharmaceuticals, for patients treated in European countries; Convupidiol, Alef Pharma, for patients treated in Argentina) from May 2019 to March 2024 were identified. Inclusion criteria were (1) epilepsy due to a monogenic cause (pathogenic or likely pathogenic variant, classified according to American College of Medical Genetics and Genomics criteria)^14^ and (2) a minimum follow‐up period of 3 months after initiating CBD.

Data collected included demographics, genetic variants, clinical history, intellectual disability (ID) and its severity according to the treating clinician and/or standardized assessments, seizure and epilepsy types, electroencephalographic (EEG) findings, previous/concomitant ASMs, and baseline seizure frequency (monthly seizure frequency during the 3 months prior to starting CBD). Patients were categorized into specific epilepsy syndromes by their treating epileptologist, with those not diagnosed as LGS, DS, or TSC‐epilepsy classified as “other DEE.”

### 2.2 Outcome measures

Data on seizure frequency, adverse events, and drug discontinuation were obtained from patient seizure diaries and clinical records. CBD effectiveness and safety were assessed at 3, 6, 12, and 24 months after CBD initiation, with follow‐up truncated at 24 months.

Effectiveness outcomes included mean seizure reduction, ≥50% seizure reduction, and seizure freedom at the last follow‐up visit relative to the baseline observation period. Mean seizure reduction at 12 and 24 months (for patients with available follow‐up) relative to the baseline observation period was also considered. Additionally, the occurrence of >25% increase in monthly seizure frequency (seizure worsening) during follow‐up compared with the baseline observation period was evaluated. Safety and tolerability outcomes included CBD retention and the incidence of side effects attributed to CBD by participating physicians.

The Clinical Global Impression (CGI) scale was used retrospectively from patient records and clinical evaluations to assess overall efficacy and tolerability, specifically, the CGI Improvement (CGI‐I) subitem.^15^ The CGI‐I asks clinicians to evaluate the degree of improvement or worsening at the end of treatment, compared with baseline, using a 7‐point Likert scale ranging from 1 (very much improved) to 7 (very much worsened). In cases with limited information, clinicians were instructed not to assign a CGI score.

### 2.3 Statistical analysis

Descriptive statistical methods and data visualization were used to assess data distribution. All statistical analyses were performed using complete case analysis, and the rate of missing data for each variable is shown in Figure S1 in Appendix S1. Comparisons among categorical variables were performed using the chi‐squared test, whereas comparisons between continuous variables were conducted with either the *t*‐test or the Mann–Whitney *U*‐test, depending on whether the distribution was normal or nonnormal, respectively.

To assess the predictors of CBD effectiveness, a general linear mixed model was utilized, incorporating follow‐up as a function of center as a random effect to account for potential heterogeneity between sites. The model included as prespecified fixed effects variables the age at seizure onset and CBD initiation, indication type (approved vs. off‐label), severity of ID, family history of epilepsy, duration of seizure freedom prior to CBD initiation, number of ASMs tried before CBD, clobazam (CLB) use, initial CBD dose, history of generalized paroxysmal fast activities on EEG, and history of tonic seizures.^7^, ^16^, ^17^, ^18^, ^19^

Additionally, to evaluate the differential effectiveness of CBD across different epilepsy syndromes (TSC‐epilepsy vs. DS vs. LGS vs. other DEE), the same model was employed, incorporating as fixed effects epilepsy syndromes and the variables identified as independently associated with treatment response in the previous model.

Statistical analyses were conducted using R Studio version 4.2.3. Test results with *p* < .05 were considered statistically significant.

## 3 RESULTS

### 3.1 Clinical and genetic features of study population

**FIGURE 1:**
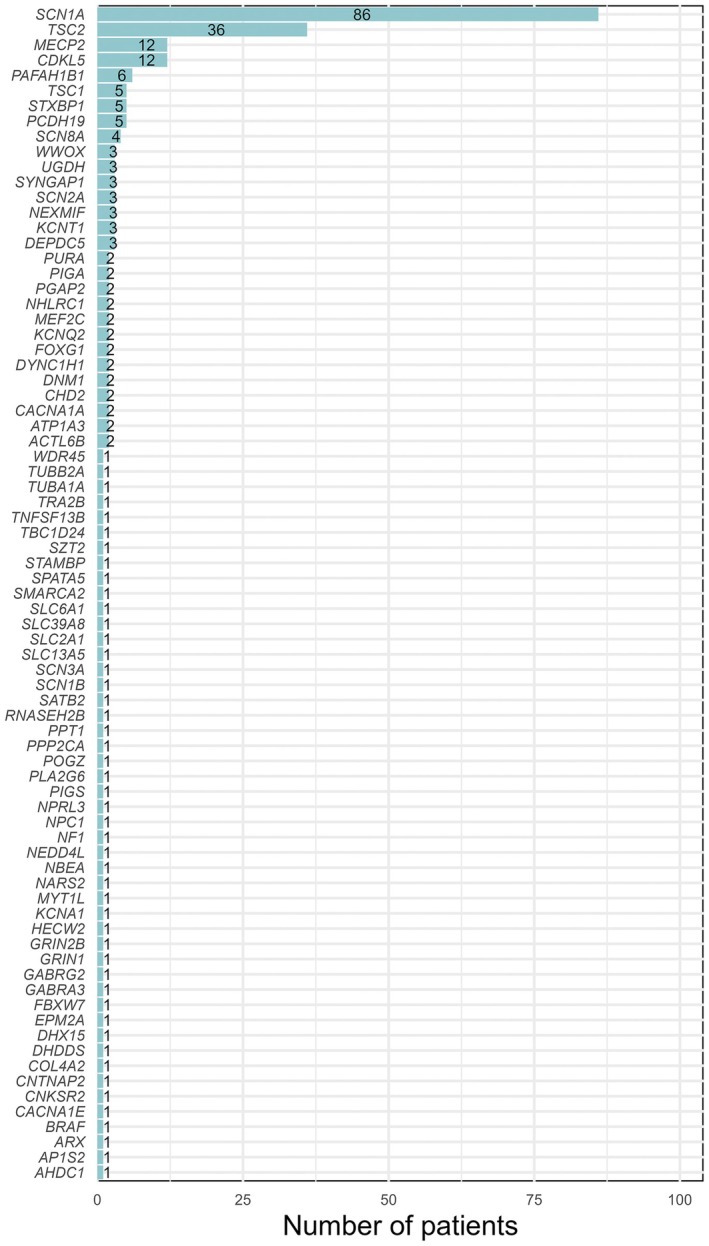
Distribution of patients by their monogenic epilepsy etiologies. Each bar represents a specific genetic cause, with the corresponding number of patients.

A total of 266 patients (135 females, 50.8%) with monogenic epilepsies receiving highly purified CBD (228 Epidiolex and 38 Convupidiol) for the treatment of drug‐resistant seizures were included based on the study criteria. The most common causative genes were *SCN1A*, found in 86 patients (32.3%), *TSC2* in 36 patients (13.5%), *CDKL5* and *MECP2*, each found in 12 patients (4.5%), *PAFAH1B1* in six patients (2.2%), and *STXBP1*, *PCDH19*, and *TSC1*, each in five patients (1.9%). Overall, 77 different monogenic epilepsies have been included; a detailed list is shown in Figure 1.

The median age at epilepsy onset was 5 months (interquartile range [IQR] = 3–14.5, range = 0–264). Patients were diagnosed by the treating clinicians with DS (86 [32.3%] patients), TSC‐epilepsy (41 [15.4%] patients), LGS (34 [12.8%] patients), progressive myoclonic epilepsy (PME; four [1.5%] patients), and other DEEs (101 [38%] patients). In the “other DEE” group, seven patients were diagnosed with Rett syndrome, four with infantile epileptic spasm syndrome (IESS), three with epilepsy of infancy with migrating focal seizures (EIMFS), and three with DEE with spike–wave activation in sleep (DEE‐SWAS).^20^, ^21^ Baseline characteristics of the study cohort are fully summarized in Table 1.

**TABLE 1:** Demographic and clinical characteristics.

Characteristic	Value
Age at epilepsy onset, months, median (IQR)	5 (3–14.5)
Sex, female, *n* (%)	135 (50.8)
Family history of epilepsy in 1st or 2nd degree relatives, *n* (%)	54 (21.5)
Intellectual disability, *n* (%)	
No	8 (3.1)
Mild	19 (7.4)
Moderate	70 (27.1)
Severe/profound	161 (62.4)
History of febrile seizures, *n* (%)	75 (29.9)
History of photosensitivity, *n* (%)	24 (9.8)
History of spike–slow wave discharges, *n* (%)	138 (56.6)
History of generalized paroxysmal fast activity, *n* (%)	53 (22)
Age at CBD prescription, years, median (IQR)	12 (7–19)
Previous history of epilepsy surgery, *n* (%)	10 (4.1)
History of vagus nerve stimulation, *n* (%)	
No	201 (82.4)
Yes but not active at CBD prescription	13 (5.3)
Yes and active at CBD prescription	30 (12.3)
ASMs tried prior to CBD prescription, *n*, median (IQR)	7 (4–9)
Seizure types experienced at CBD prescription, *n* (%)	
Tonic seizures	118 (44.5)
Atonic seizures	32 (12.1)
Focal seizures	102 (38.5)
Absence seizures	66 (24.9)
Generalized tonic–clonic seizures	153 (57.7)
Myoclonic seizures	86 (32.5)
Epileptic spasms	49 (18.5)

Abbreviations: CBD, cannabidiol; IQR, interquartile range.

### 3.2 ASM data and response to treatment

The median age at the time of CBD initiation was 12 years (IQR = 7–19), with a mean (SD) initial target daily dose of 8.5 mg/kg/day (±4.6) and a median follow‐up duration of 17 months (IQR = 12–24) after CBD initiation.

**FIGURE 2:**
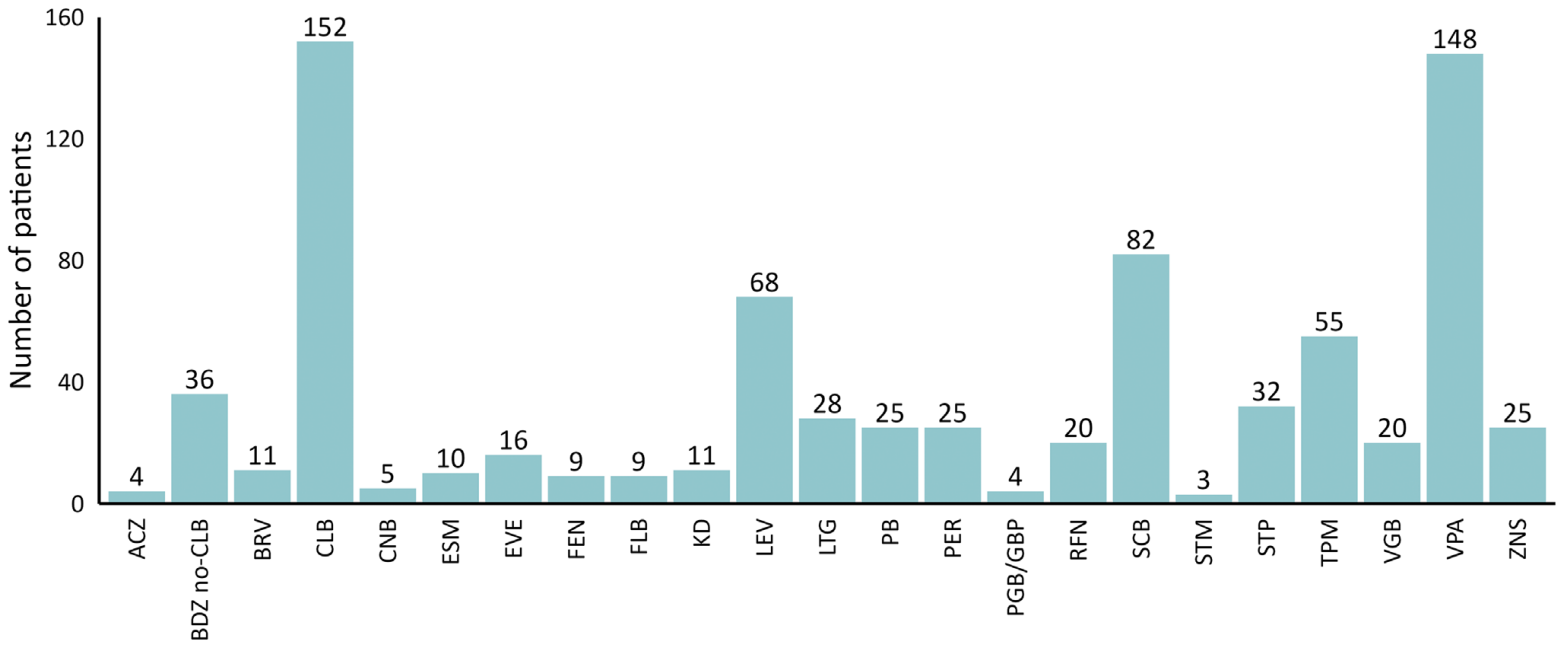
Antiseizure medications used at the time of cannabidiol prescription. Bars show the distribution of antiseizure medications (ASMs) used at the time of cannabidiol treatment initiation, with the number of patients per ASM indicated. Legend: ACZ: acetazolamide; BDZ: benzodiazepines; BRV: brivaracetam; CLB: clobazam; CNB: cenobamate; ESM: ethosuximide; EVE: everolimus; FEN: fenfluramine; FLB: felbamate; KD: ketogenic diet; LEV: levetiracetam; LTG: lamotrigine; PB: phenobarbital: PER: perampanel; PGB: pregabalin; GBP: gabapentin; RFN: rufinamide; SCB: sodium channel blockers; STM: Sulthiame STP: stiripentol; TPM: topiramate; VGB: vigabatrin VPA: valproic acid; ZNS: zonisamide.

At the time of CBD initiation, patients were taking a median of 3 ASMs (IQR = 2–4), and 11 were on a ketogenic diet (Figure 2). The median longest period of remission prior to CBD initiation was 7 days (IQR = 0–25), with 57.1% of patients having never experienced >7 days of remission during their epilepsy history. Regarding seizure frequency, 153 (59.8%) patients experienced daily seizures, 58 (22.7%) weekly seizures, 41 (15.4%) monthly seizures, and four (1.5%) less frequent seizures. Among seizure types reported at the time of CBD initiation, the most common was generalized tonic–clonic seizures in 157 (57.7%) patients, followed by tonic seizures in 118 (44.5%; Table 1).

The mean seizure reduction across the study population at the last follow‐up visit was 38.6% (95% confidence interval [CI] = 34.5–42.7). For patients with available 12‐month (174 patients) and 24‐month (80 patients) follow‐up data, the mean seizure reduction was 40.5% (95% CI = 35.6–45.3) and 39.7% (95% CI = 32.7–46.7), respectively. A total of 123 (47.5%) patients achieved ≥50% seizure reduction at the last visit, whereas 56 (21.6%) patients achieved ≥75% reduction, and 19 (7.4%) patients achieved seizure freedom. Conversely, seizure worsening was observed in 15 patients (5.6%) without a clear association with specific genetic etiologies or distinctive demographic, clinical, or treatment characteristics. In particular, no significant differences were found regarding sex, epilepsy syndrome, median duration of seizure remission, age and frequency of seizures at CBD initiation, daily target dose of CBD, or the number of prior ASMs used. Regarding ID severity, it is worth mentioning that 15 of 15 patients experiencing seizure worsening had severe ID, albeit this difference did not reach any statistical significance. See Supplementary Results in Appendix S1 for a detailed list of variants harbored by these patients along with their clinical characteristics.

Regarding the CGI‐I scale, improvement was observed in 150 patients (65.8%), with 21 of these (14%) reported as very much improved, 61 (40.7%) as much improved, and 68 (45.3%) as slightly improved. In contrast, 19 patients (8.3%) were reported to have worsened with CBD treatment, and 59 patients (22.2%) experienced no change in their overall condition (see Supplementary Results in Appendix S1 for a detailed list of variants of patients experiencing worsening based on CGI‐I). Finally, ASM retention at the last follow‐up was seen in 221 (84.4%) patients.

**FIGURE 3:**
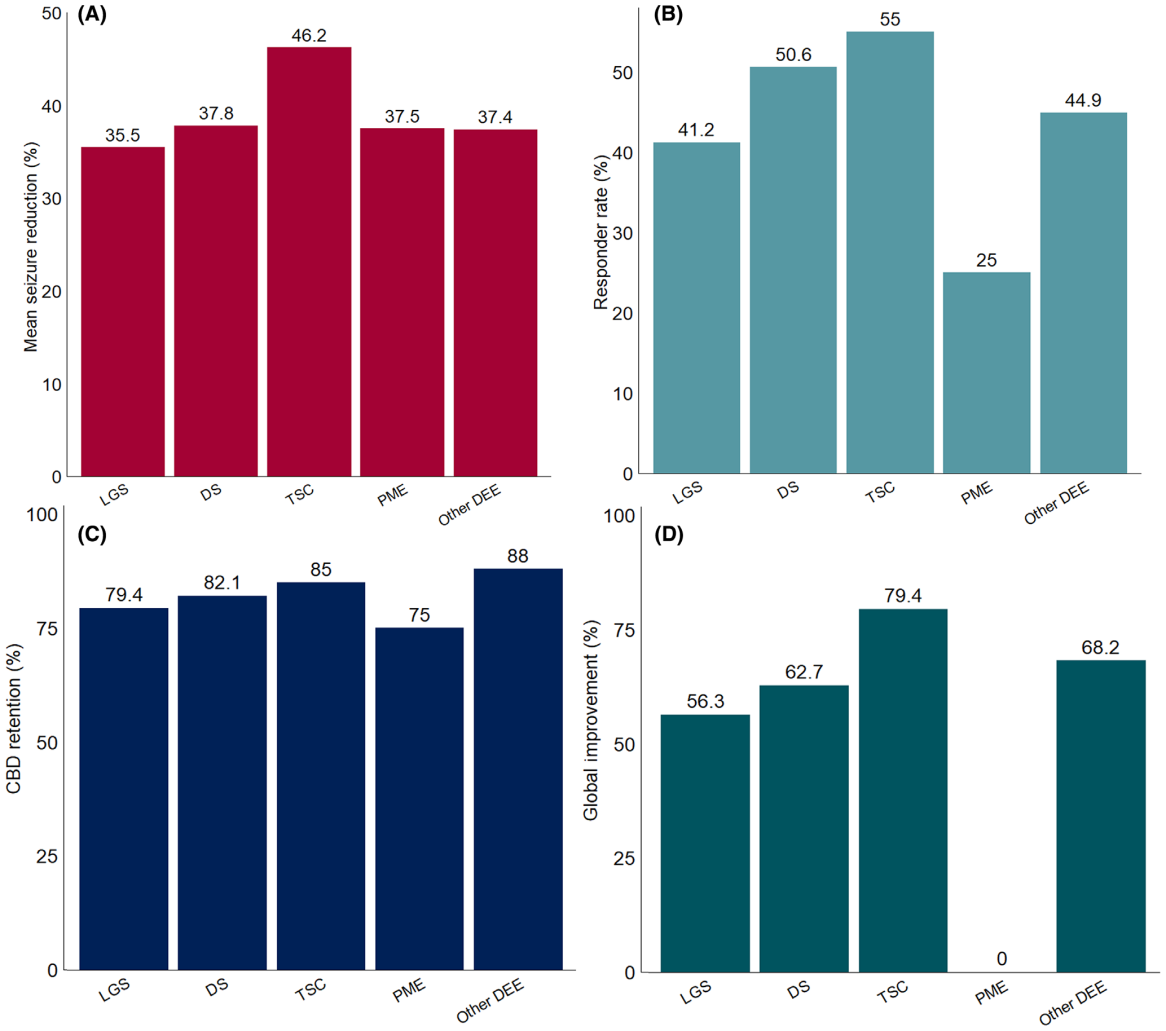
Response to cannabidiol (CBD) treatment at the last visit stratified by epilepsy syndrome. (A) Mean percentage reduction in seizure frequency from baseline at the last visit. (B) Proportion of patients achieving ≥50% seizure reduction (responder rate). (C) Retention rate, indicating continued CBD use until the last visit. (D) Clinical Global Impression improvement, showing the percentage of patients rated as improved by the treating epileptologist. DEE, developmental and epileptic encephalopathy; DS, Dravet syndrome; LGS, Lennox–Gastaut syndrome; PME, progressive myoclonic epilepsy; TSC, tuberous sclerosis complex.

When examining epilepsy syndromes for which CBD was prescribed by the treating physician—specifically LGS, DS, TSC‐epilepsy, and other DEE—no significant differences among groups were observed across mean seizure reduction, ≥50% responder rates, CGI improvement, and CBD retention rate at the last follow‐up (*p* > .4 for all comparisons). Conversely, CGI‐I improvement was significantly lower in patients with PME compared with other epilepsy syndromes (0% vs. 66.4%, *p* = .049). Detailed results for each outcome measure stratified by epilepsy syndrome are outlined in Figure 3. Within the “other DEE” group, the mean (SD) seizure reduction and the proportion of patients achieving ≥50% seizure reduction were 37.5% (±32.3) and 50% for IESS, 36.7% (±32.1) and 66.7% for EIMFS, and 53.3% (±5.8) and 100% for DEE‐SWAS, respectively.

When assessing the concordance between ≥50% seizure reduction and CGI‐I improvement from baseline, a moderate agreement was found. Specifically, 96 of 107 (89.7%) patients classified as responders also showed CGI‐I improvement, although CGI‐I improvement was also observed in 51 of 115 (44.3%) patients not considered responders (kappa = .44, *p* < .001).

### 3.3 Response to treatment according to specific genes

When considering genes with at least five patients treated with CBD, *TSC2* and *PAFAH1B1* had the highest 50% seizure reduction rates, whereas the highest rate of CGI‐I improvement from baseline was reported in *TSC2* and *MECP2*. A detailed representation of different outcome measures across all genes with at least two patients treated with CBD is presented in Table 2, whereas response to treatment in the entire population according to gene etiology is further detailed in Table S1 in Appendix S1.

**TABLE 2:** Response to treatment according to gene etiology (including genes with ≥2 pts treated with CBD).

Gene	Mean seizure reduction, % (SD)	50% responder rate	CGI improvement
*SCN1A* (86 pts)	37.6 (33)	50.6%	63.9%
*TSC2* (36 pts)	48.4 (38.1)	55.9%	83.3%
*MECP2* (12 pts)	30.9 (32.1)	33.3%	77.8%
*CDKL5* (12 pts)	27.1 (33.3)	33.3%	63.6%
*PAFAH1B1* (6 pts)	45.8 (40.5)	60%	60%
*TSC1* (5 pts)	21.4 (29.5)	2/5	2/4
*STXBP1* (5 pts)	31 (32.5)	2/5	1/3
*PCDH19* (5 pts)	36.6 (41.6)	2/5	4/5
*SCN8A* (4 pts)	20 (40)	1/4	2/4
*WWOX* (3 pts)	41.7 (38.2)	2/3	2/3
*UGDH* (3 pts)	30 (26.4)	1/3	1/3
*SYNGAP1* (3 pts)	54 (5.6)	3/3	2/3
*SCN2A* (3 pts)	36.7 (32.1)	2/3	2/3
*NEXMIF* (3 pts)	45.6 (45.1)	2/3	3/3
*KCNT1* (3 pts)	23.3 (20.8)	0	2/3
*DEPDC5* (3 pts)	51.7 (45.4)	1/3	1/3
*PURA* (2 pts)	45 (21)	1/2	1/2
*PIGA* (2 pts)	64.3 (50.5)	1/2	2/2
*PGAP2* (2 pts)	40 (28.2)	1/2	2/2
*NHLRC1* (2 pts)	25 (7.1)	0	0
*MEF2C* (2 pts)	21.5 (30.4)	0	0
*KCNQ2* (2 pts)	37.5 (53)	1/2	1/2
*FOXG1* (2 pts)	89.5 (7.8)	2/2	2/2
*DYNC1H1* (2 pts)	45 (21.2)	1/2	2/2
*DNM1* (2 pts)	0 (0)	0	0
*CHD2* (2 pts)	92.5 (10.6)	2/2	2/2
*CACNA1A* (2 pts)	50 (0)	2/2	1/2
*ATP1A3* (2 pts)	10 (14.1)	0	1/2
*ACTL6B* (2 pts)	0 (0)	0	0

*Note*: Percentages have been reported only for groups with more than five patients.

Abbreviation: CGI, Clinical Global Impression; pts, patients.

When grouping genes into well‐defined functional categories, a ≥50% seizure reduction was achieved in nine of 18 (50%) non‐S*CN1A* channelopathies. Specifically, mean seizure reduction (SD) and ≥50% seizure reduction were 50% (±0) and 100% for calcium channel genes (*n* = 3), 32.5% (±29.3) and 33.3% for potassium channel genes (*n* = 6), and 33.3% (±35) and 44.4% for non‐*SCN1A* sodium channel genes (*n* = 9), respectively. Regarding genes involved in γ‐aminobutyric acid (GABA; *n* = 3; *SLC6A1*, *GABRA3*, *GABRG3*) and glutamatergic transmission (*n* = 5; *SYNGAP*, *GRIN1*, *GRIN2B*), mean seizure reduction and ≥50% seizure reduction were 8.3% (±14.4) and 0% for GABA‐related genes (*n* = 3), and 29.5% (±28.8) and 50% for glutamate receptor genes. Considering non‐TSC genes pertaining to the mTOR pathway (i.e., *DEPDC5*, *NPRL3*, *SZT2*; *n* = 5), the mean seizure reduction was 45% (±37.7), with ≥50% seizure reduction occurring in 40% of patients. Finally, for genes associated with Rett and Rett‐like syndromes (namely, *MECP2*, *CDKL5*, *FOXG1*, *MEF2C*; *n* = 28 patients), the mean seizure reduction was 32.8% (±34.2), and ≥50% seizure reduction was observed in 35.7% of patients.

### 3.4 Predictors of treatment response

When comparing approved and off‐label prescriptions (i.e., LGS, DS, and TSC‐epilepsy vs. others), no significant differences emerged in terms of seizure reduction (label 39.1%, 95% CI = 33.6–44.5 vs. off‐label 37.4%, 95% CI = 31.1–43.6; *p* = .69), ≥50% responder rate (49.4%, 95% CI = 41.4%–57.3% vs. 44.1%, 95% CI = 34.3–53.9; *p* = .41), and CGI‐I improvement (65.2%, 95% CI = 57.3–73.2 vs. 66.7%, 95% CI = 56.6–76.8; *p* = .83).

In the general linear mixed model of seizure reduction using recruiting center as random effect, the only variables independently associated with reduced CBD effectiveness were a shorter maximum duration of seizure freedom in days before CBD initiation (*p* = .029) and a higher severity of ID compared to normal/borderline intellectual functioning (mild ID, *p* = .04, moderate/severe ID *p* = .026). No significant differences were found based on the concomitant prescription of CLB (*p* = .9), nor based on approved or off‐label prescription (*p* = .25; see Table S2 in Appendix S1 for full model results).

When specifically examining the effect of epilepsy diagnosis on CBD response after adjusting for ID severity and maximum duration of seizure freedom, no significant differences emerged among the main diagnostic groups (LGS vs. DS vs. TSC vs. other DEE; *p* = .83), with estimated marginal means remaining similar across groups (Figure S2 in Appendix S1).

### 3.5 Side effects

**FIGURE 4:**
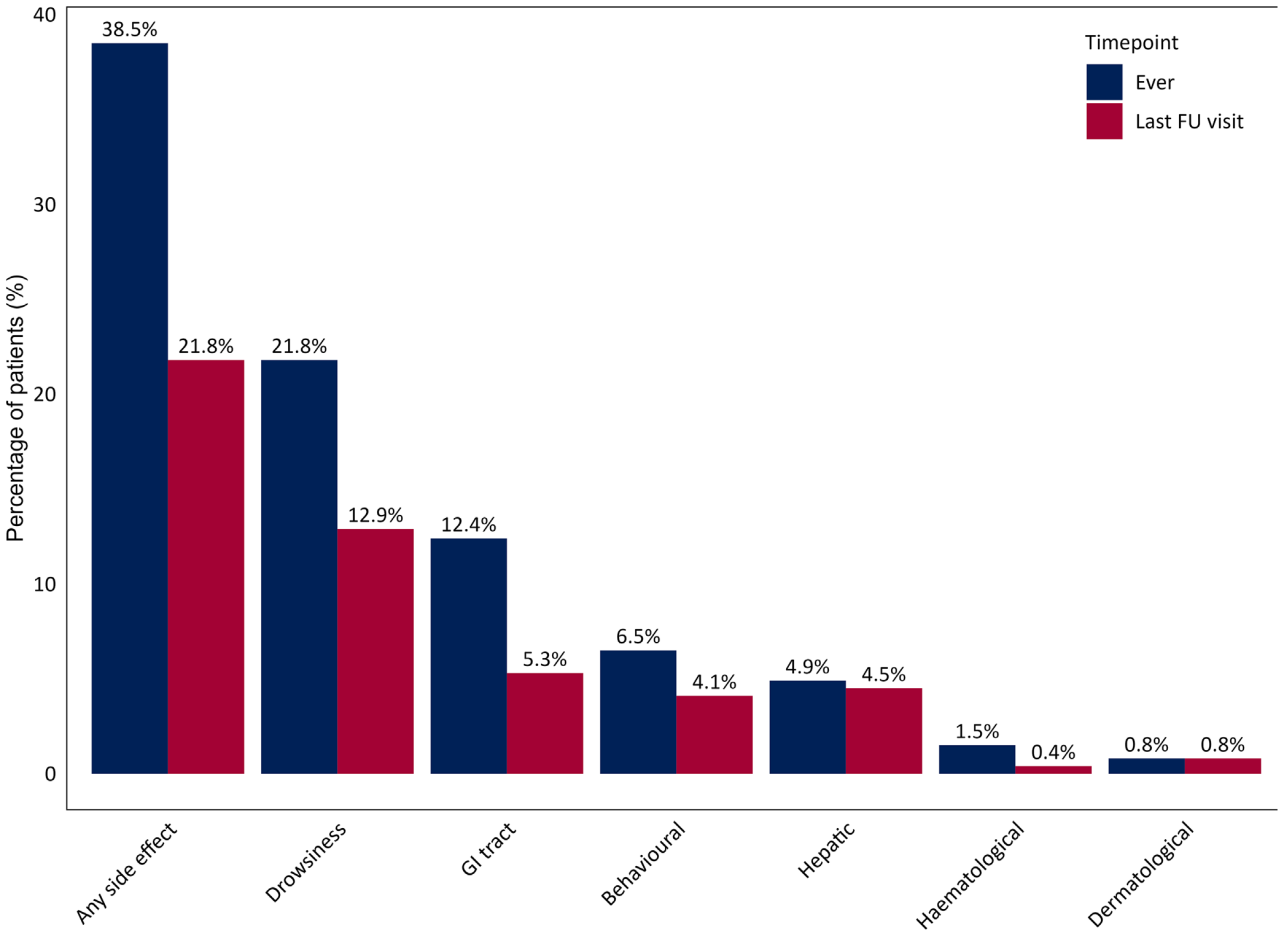
Side effects over time during cannabidiol treatment. Blue bars represent the proportion of patients reporting each side effect at any time during follow‐up, whereas red bars indicate those experiencing the same side effects at the last visit. FU, follow‐up; GI, gastrointestinal.

After the prescription of CBD, side effects during follow‐up were reported in 101 patients (38.5%). Notably, these side effects were transitory in approximately half of the patients. At the last visit, side effects were reported in 58 patients (21.8%), with drowsiness being the most common, observed in 34 patients (12.9%), followed by gastrointestinal side effects in 14 patients (5.3%). For a detailed representation of side effects during follow‐up and at the last visit, please see Figure 4.

## 4 DISCUSSION

Whereas previous RCTs have underscored the efficacy of CBD in specific DEEs and monogenic epilepsies, this study systematically evaluates its potential across a broad range of rare monogenic epilepsies. The results of this multicenter, real‐world study indicate that CBD can lead to clinically meaningful seizure reductions in a variety of monogenic epilepsies, including those beyond currently approved indications, with no significant differences observed between approved and off‐label uses.

The mean seizure reduction at the last visit was 38.6%, with responder rates of 47.5%, consistent with previous RCTs in LGS and DS but slightly slower than those reported with TSC.^4^, ^5^, ^6^ The responder rates observed in the present study should be interpreted in the context of the substantial seizure burden in our cohort. Notably, more than half of the patients had never experienced more than a week of remission before initiating CBD, and daily or multiple daily seizures were common in most cases. This pronounced epilepsy severity was somewhat expected, especially in the “other DEE” group, where off‐label CBD prescriptions were likely used in more refractory cases following repeated failure of approved treatment strategies. Accordingly, when reviewing possible predictors of CBD response, we identified shorter maximum seizure‐free duration before CBD initiation and higher ID levels—both proxies for underlying epilepsy severity—as the primary factors associated with less favorable outcomes.^19^ Notably, TSC patients exhibited high seizure reduction and overall improvement on the CGI‐I scale, confirming the notable effectiveness of CBD in this group.^6^, ^22^ However, it is worth noting that TSC cases might include both DEEs and focal epilepsies, with the latter potentially explaining the higher response rate due to lower epilepsy severity. When accounting for this confounding factor, the estimated mean seizure reduction was comparable across TSC and other diagnostic groups, including patients with other DEEs. Additionally, the general linear mixed model did not identify approved versus off‐label indications as significant predictors of seizure reduction, supporting the potential for expanding CBD indications beyond currently approved conditions, aligning with recent evidence from a real‐world study in patients with diverse epilepsy etiologies.^23^ Similarly, the multivariable analysis failed to identify a differential effectiveness of CBD based on concomitant CLB use, consistent with the same real‐world study by Kühne and colleagues.^23^

An additional insight from this study derived from the assessment of CBD effectiveness across specific monogenic epilepsies and gene functional groups. Beyond *TSC2* and *SCN1A* patients, who, as expected, demonstrated high seizure reduction rates, other monogenic epilepsies also showed favorable responses. Although based on a small sample of six patients, the favorable effectiveness profile of CBD in individuals with *PAFAH1B1* pathogenic variants, typically associated with malformations of cortical development, suggests a potential role for CBD in epilepsies with combined structural and genetic etiologies, similar to its observed effects in TSC. Prior evidence has highlighted the role of neuroinflammation in treatment‐refractory seizures linked to structural lesions, particularly in cortical tubers and focal cortical dysplasia.^24^, ^25^ Considering the possible impact of CBD on neuroinflammation,^26^, ^27^ we cannot exclude that this mechanism may partially contribute to its positive therapeutic effects in these epilepsies. Similarly, the anti‐inflammatory properties of CBD may help explain the excellent response observed in patients with DEE‐SWAS, where neuroinflammation has been repeatedly proposed as a contributing factor.^28^ Although our findings are limited to only three patients, they align with a recent case report by Ferrera et al. and may encourage future studies specifically investigating the potential effectiveness of CBD in this rare DEE.^29^

The hypothesized antiseizure effects of CBD have been primarily linked to desensitization of TRPV (transient receptor potential vanilloid) and functional antagonism of GPR55 (G protein‐coupled receptor‐55), ultimately stabilizing intracellular Ca^2+^ levels and modulating neuronal excitability.^30^, ^31^ Similarly, previous research indicates that CBD may influence calcium currents through interactions with T‐type Ca^2+^ channels.^31^ Although this study included only three patients with pathogenic calcium channel variants, all responded to CBD. Although this finding is limited by the small sample size, it may warrant further investigation into potential calcium‐related mechanisms underlying the efficacy of CBD in epilepsy.

Another interesting finding was the varying effectiveness of CBD among patients with gene mutations affecting GABAergic and glutamatergic transmission. Among patients with pathogenic variants in genes related to glutamate signaling, all three cases with *SYNGAP1* variants exhibited an optimal seizure response, aligning with recent findings by Kuchenbuch and colleagues and supporting the potential role of CBD in the treatment of this specific DEE.^8^ Conversely, none of the patients with mutations directly linked to GABAergic transmission (e.g., *SLC6A1*, *GABRA3*, *GABRG3*) responded to CBD, suggesting that genetic disruptions in GABAergic signaling might negatively impact CBD effectiveness.

Importantly, although CBD treatment led to meaningful seizure reduction, the moderate concordance between seizure reduction and CGI‐I scores suggests that CBD may have effects beyond seizure control. Although nearly all patients who responded to CBD were reported to have a global improvement, approximately half of those who did not respond to CBD in terms of seizure control still reported improvement in global clinical status. This may align with recent literature indicating a positive effect of CBD on various nonseizure outcome measures, including cognitive abilities, quality of life, sleep, motor function, and mood.^32^, ^33^, ^34^ The low concordance between seizure reduction and CGI‐I improvement was particularly evident in patients with *MECP2* and *CDKL5* disorders, where relatively modest seizure reduction was coupled with high rates of CGI‐I improvement, in line with recent findings highlighting the positive effects of CBD on psychiatric and motor symptoms in patients with Rett syndrome.^9^ However, given the potential limitations of CGI‐I as a subjective assessment tool, future studies should incorporate additional validated instruments—such as quality‐of‐life scales, neurodevelopmental assessments, and caregiver‐reported outcomes—to comprehensively evaluate the potential impact of CBD beyond seizure frequency.

This study also found that CBD was generally well tolerated, with high retention rates at the last visit. Although side effects were reported in approximately 40% of patients, these were transient in about half of the cases, with drowsiness/sedation and gastrointestinal side effects being the most frequently observed, confirming previous findings.^35^ Additionally, only a minority of patients (5.6%) experienced seizure worsening, with proportions similar to those highlighted in previous RCTs, supporting the safety of CBD across a broad group of patients with rare genetic epilepsies.

Finally, although this study predominantly included patients with DEEs, it also incorporated four patients with PME attributed to distinct genetic variants. Within this small subgroup, the response to CBD was modest; only one patient achieved a 50% reduction in seizures, and none demonstrated improvements on the CGI‐I scale. These findings suggest that the off‐label application of CBD in this particular patient population may have limited therapeutic value.

Despite the strengths of this study, including a large multicenter cohort with a genetic etiology and the inclusion of patients carrying confirmed pathogenic or likely pathogenic variants, some limitations must be acknowledged. First, its retrospective design inherently introduces potential recall bias and heterogeneity in data collection across multiple centers. Future prospective studies with standardized protocols, including seizure diaries and structured cognitive and behavioral assessments, will be crucial in validating our findings. Additionally, the absence of standardized questionnaires for assessing side effects at different timepoints during treatment duration may have led to their underestimation compared to previous findings from RCTs. Lastly, the small number of patients with individual genetic etiologies limited the ability to evaluate the effectiveness of CBD across specific monogenic epilepsies. In particular, in smaller subgroups with only two or three patients, the observed response rates may be influenced by chance or individual patient characteristics rather than the underlying genetic cause. Given the rarity of many monogenic epilepsies, large‐scale collaborations across international epilepsy centers and multi‐institutional registries with dedicated genetic subgroups are needed to further investigate differential responses to CBD across diverse genetic etiologies.

In conclusion, this study's findings highlight the potential role of CBD as an effective treatment option for patients with monogenic epilepsies, extending beyond previously approved conditions. Notably, we observed no significant differences in effectiveness between approved and off‐label uses, with promising results in monogenic DEEs beyond LGS, TSC, and DS. The real‐world effectiveness and safety observed in this study provide valuable insight for clinicians treating patients with rare genetic epilepsies, who frequently face limited treatment options and may often need to rely on off‐label therapies. Lastly, preliminary findings from small patient subgroups suggest that certain monogenic epilepsies may respond particularly well to CBD, emphasizing the need for large registries and prospective studies to confirm these results and potentially expand their therapeutic indications.

## AUTHOR CONTRIBUTIONS

ECI contributed to drafting a significant portion of the manuscript and figures. ECI, AO, AC, contributed to the concept and design of the study. ECI, AM, RHC, MP, PBM, JPC, MR, KMM, AS, LL, VDG, GRV, SC, EC, FFO, JDC, ARC, AF, GS, AAS, ARu, MMM, GP, ARi, LB, DMC, PB, FB, GDG, SM, PS, ND, CM, DB, CDB, GR, EG, AO, AC significantly contributed to the acquisition and analysis of the data and critically revised the manuscript for intellectual content. GENE‐CBD study group: Significant contribution to data acquisition.

## CONFLICT OF INTEREST STATEMENT

E.C.I. reports speaking honoraria from Lusofarmaco, outside the submitted work. P.B.M. has served on an advisory board for Angelini Pharma and has received travel expenses from UCB Pharma, outside the submitted work. J.P.‐C. has served as a speaker and has received travel expenses from Angelini Pharma and LivaNova, outside the submitted work. V.D.G. has served on scientific advisory boards for Longboard Pharmaceuticals, has received research grants from Jazz Pharmaceuticals, and has received speaker and consultancy fees from Jazz Pharmaceuticals, Novartis, Nutricia, Vitaflo, and Dr. Schar Kanso, outside the submitted work. J.D.‐C. has received speaker honoraria from Jazz Pharmaceuticals, outside the submitted work. F.B. has served on scientific advisory boards for Angelini Pharma, UCB, Jazz Pharmaceuticals, Ethypharm, and Eisai and has received speaker honoraria from Angelini Pharma and UCB, outside the submitted work. Her research receives support from the Multidisciplinary Transition Model for Patients With Complex Epilepsy (Trans‐Epic) project, supported by Ricerca Corrente. S.M. has served on scientific advisory boards for Zogenix, UCB, and Ethypharm and has received speaker honoraria for Biocodex, Jazz Pharmaceuticals, Eisai, and UCB, outside the submitted work. N.D. has served as a paid advisor and/or speaker for Actiobio Sciences, Angelini Pharma, Bial, Eisai, Jazz Pharmaceuticals, LivaNova, and UNEEG Medical, outside the submitted work. C.D.B. reports personal fees from UCB Pharma, Eisai, Jazz Pharmaceuticals, Angelini Pharma, Lusofarmaco, and Ecupharma, outside the submitted work. G.R. has served on advisory boards or received speaker honoraria paid to her department from Angelini, Bial, Jazz Pharmaceuticals, Neuraxpharm, Neurocrine, and Takeda, outside the submitted work. Her research receives support from the Swiss National Science (SNSF: 208184) and Anna Mueller Grocholski Foundations. The other authors report no disclosures relevant to this article. We confirm that we have read the Journal's position on issues involved in ethical publication and affirm that this report is consistent with those guidelines.

## Supporting information


Appendix S1.

## Data Availability

Anonymized data will be available to qualified academic investigators to replicate study results and as long as data transfer is in agreement with EU legislation regarding general data protection regulation. Data transfer will be regulated by material transfer agreements and should be authorized by institutional review boards.

## Appendix

Coinvestigators in the GENE‐CBD (Genetic Epilepsy Network for Cannabidiol Effectiveness) Study Group: Alessandra Morano, Department of Human Neurosciences, Sapienza University, Rome, Italy, site investigator, data acquisition; Nicola Simeone, Department of Neuroscience, Reproductive Sciences and Odontostomatology, Federico II University of Naples, Naples, Italy, site investigator, data acquisition; Claudia Cuccurullo, AORN Cardarelli, Naples, Italy, site investigator, data acquisition; Massimo Scacciati, Department of Clinical and Experimental Medicine, University of Pisa, Pisa, Italy, site investigator, data acquisition; Alice Bonuccelli, Department of Clinical and Experimental Medicine, University of Pisa, Pisa, Italy, site investigator, data acquisition; Maddalena Bianchetti, Pediatric Neurology and Psychiatric Unit, Fondazione Policlinico Universitario Agostino Gemelli IRCCS, Rome, Italy, site investigator, data acquisition; Alberto Verrotti, Department of Medical and Surgical Sciences, Pediatric Clinic, University of Perugia, Perugia, Italy, site investigator, data acquisition; Marta Piccioli, UOC Neurology, PO San Filippo Neri, ASL Roma 1, Rome, Italy, site investigator, data acquisition; Massimiliano Celario, Brain and Behavioral Sciences Department, University of Pavia, Pavia, Italy; Childhood and Adolescence Epilepsy Center, Department of Child Neurology and Psychiatry, IRCCS Mondino Foundation, European Reference Network (ERN) for Rare and Complex Epilepsies (EpiCARE) full member, Pavia, Italy, site investigator, data acquisition; Elena Tartara, Epilepsy Center, IRCCS Mondino Foundation, ERN EpiCARE full member, Pavia, Italy, site investigator, data acquisition; Giulia Balletto, Department of Neuroscience, Rehabilitation, Ophthalmology, Genetics, Maternal and Child Health (DINOGMI), University of Genoa, Genoa, Italy, Site investigator, Data acquisition; Chiara Bagliani, Department of Neuroscience, Rehabilitation, Ophthalmology, Genetics, Maternal and Child Health, University of Genoa, Genoa, Italy, site investigator, data acquisition; Beatriz Gonzalez Giraldez, Epilepsy Unit, Neurology Service, Hospital Universitario and IIS Fundación Jiménez Díaz and CIBERER, Madrid, Spain, site investigator, data acquisition; Pasquale Parisi, Department of Neuroscience, Mental Health, and Sense Organs, Faculty of Medicine and Psychology, Sant'Andrea Hospital, Rome, Italy, site investigator, data acquisition; Carmen Salluce, IRCCS Stella Maris Foundation, Pisa, Italy; University of Pisa, Pisa, Italy, site investigator, data acquisition; Emanuele Bartolini, IRCCS Stella Maris Foundation, Pisa, Italy; University of Pisa, Pisa, Italy, site investigator, data acquisition; Maria Del Carmen Fons‐Estupiña, Pediatric Neurology Department, Institut de Recerca Sant Joan de Déu, Sant Joan de Déu University Hospital, Barcelona, Spain, site investigator, data acquisition; Federico Ramos, Department of Child Neurology, Epilepsy and Neurophysiology Unit, member of the ERN EpiCARE, Hospital Sant Joan de Dèu, Barcelona, Spain, site investigator, data acquisition; Alberto Danieli, Scientific Institute IRCCS E. Medea, Epilepsy and Clinical Neurophysiology Unit, Conegliano, Italy, site investigator, data acquisition; Alessandra Rossi, Scientific Institute IRCCS E. Medea, Epilepsy and Clinical Neurophysiology Unit, Conegliano, Italy, site investigator, data acquisition; Elisabetta Pantaloni, Child Neurology and Psychiatric Unit; Pediatric Hospital G. Salesi; Azienda Ospedaliero‐Universitaria delle Marche, Ancona, Italy, site investigator, data acquisition; Ana Laura Fernandez‐Perrone, Epilepsy and Neurogenetics Unit, Vithas la Milagrosa University Hospital, Vithas Hospital Group, Madrid, Spain, site investigator, data acquisition; Giorgio Magli, Department of Woman's and Child's Health, Child Neuropsychiatry Unit, Center of Pediatric Epilepsies, AOU Sassari, University of Sassari, Sassari, Italy, site investigator, data acquisition; Ilaria Onida, Department of Woman's and Child's Health, Child Neuropsychiatry Unit, Center of Pediatric Epilepsies, AOU Sassari, University of Sassari, Sassari, Italy, site investigator, data acquisition; Giulia Bruschi, Department of Biomedical and Neuromotor Sciences, University of Bologna, Bologna, Italy, site investigator, data acquisition; Lorenzo Muccioli, Department of Biomedical and Neuromotor Sciences, University of Bologna, Bologna, Italy; IRCCS, Istituto delle Scienze Neurologiche di Bologna, full member of the ERN EpiCARE, Bologna, Italy, site investigator, data acquisition; Tomasz S. Mieszczanek, Danish Epilepsy Center, Dianalund, Denmark, member of the ERN EpiCARE, site investigator, data acquisition; Trine Hammer, Danish Epilepsy Center, Dianalund, Denmark, member of the ERN EpiCARE, site investigator, data acquisition; Francesca Furia, Danish Epilepsy Center, Dianalund, Denmark, member of the ERN EpiCARE; University of Southern Denmark, Odense, Denmark, site investigator, data acquisition.
