# Supplementary material for: Expanding the therapeutic role of highly purified cannabidiol in monogenic epilepsies: A multicenter real‐world study
Source: Epilepsia. 2025 Mar 24;66(7):2253–67. doi: 10.1111/epi.18378 (PMC12291005; doi:10.1111/epi.18378)

Expanding the Therapeutic Role of Highly Purified Cannabidiol in Monogenic Epilepsies: A Multicenter Real-World Study

Emanuele Cerulli Irelli,[1] Adolfo Mazzeo,[1] Roberto H Caraballo,[2] Marco Perulli,[3] Patrick B. Moloney,[4] Javier Peña-Ceballos,[5] Marica Rubino,[6] Katarzyna M Mieszczanek,[7] Andrea Santangelo,[8] Laura Licchetta,[9] Valentina De Giorgis,[10] Gabriela Reyes Valenzuela,[2] Susanna Casellato,[12] Elisabetta Cesaroni,[13] Francesca F Operto,[14] Jana Dominguez Carral,[15] Alia Ramírez Camacho,[15] Alessandro Ferretti,[16] Giuseppe Santangelo,[17] Angel Aledo-Serrano,[18] Andrea Rüegger,[19] Maria M Mancardi,[20] Giulia Prato,[21] Antonella Riva,[20,21] Luca Bergonzini,[9] Duccio M Cordelli,[9] Paolo Bonanni,[22] Francesca Bisulli,[9-23] Giancarlo Di Gennaro,[24] Sara Matricardi,[25] Norman Delanty,[5,26,27] Carla Marini,[13] Domenica Battaglia,[3] Carlo Di Bonaventura,[1] Georgia Ramantani,[19] Elena Gardella,[7-28] GENE-CBD study group, Alessandro Orsini,[8]* Antonietta Coppola[6]*

* These authors contributed equally to this work

Supplementary results

The mean seizure reduction across the study population at the last follow-up visit was 38.6% (95% CI: 34.5–42.7). For patients with available 12-month (174 patients) and 24-month (80 patients) follow-up data, the mean seizure reduction was 40.5% (95% CI: 35.6–45.3) and 39.7% (95% CI: 32.7–46.7), respectively. A total of 123 (47.5%) patients achieved ≥50% seizure reduction at the last visit, while 56 (21.6%) achieved ≥75% reduction, and 19 (7.4%) patients achieved seizure freedom. Conversely, seizure worsening after cannabidiol (CBD) initiation was observed during follow-up in 15 patients (5.6%), without a clear association with specific genetic etiologies or distinctive clinical and demographic characteristics. In particular, no significant differences were found regarding sex (9/122 females [6.9%] vs. 6/129 males [4.4%], p=0.39), epilepsy syndrome (Dravet syndrome: 4/82 [4.7%] vs. Lennox-Gastaut syndrome: 2/32 [5.9%] vs. Tuberous sclerosis complex-epilepsy: 3/38 [7.3%] vs. other developmental and epileptic encephalopathies: 6/95 [5.9%], p=0.96), median duration of seizure remission (3 days [interquartile range (IQR) 0–8.5] among patients experiencing worsening vs. 7 days [IQR 0–25], p=0.12), age at CBD initiation (15 years [IQR 10–23] vs. 12 years [IQR 7–19], p=0.2), seizure frequency at CBD initiation (3/42 [6.7%] with less than monthly seizures, 3/55 [5.2%] with monthly seizures, 9/144 [5.9%] with daily seizures, p=0.95), mean (±

standard deviation) initial daily target dose of CBD in mg/kg (7.3 ± 3.6 vs 8.6 ± 4.6, p=0.32), or the number of prior antiseizure medications used (6 [IQR 5–9] vs. 7 [IQR 4–9], p=0.93). Interestingly, all 15 patients with seizure worsening had severe intellectual disability (ID), although this difference did not reach statistical significance (p=0.39). Regarding pathogenic variants of patients showing seizure worsening, this occurred in three patients harboring *TSC2* pathogenic variants, three *SCN1A*, and one each *MECP2*, *CDKL5*, *TUBB2A*, *TUBA1A*, *SCN8A*, *PLA2G6*, *NEXMIF*, *MEF2C*, and *KCNT1*.

Regarding the CGI-I scale, improvement was observed in 150 patients (65.8%), with 21 of these (14%) reported as very much improved, 61 (40.7%) as much improved, and 68 (45.3%) as slightly improved. In contrast, 19 patients (8.3%) were reported to have worsened with CBD treatment, including eight patients harboring pathogenic *SCN1A* variants, three *TSC2*, and one each *MECP2*, *SLC39A8*, *STXBP1*, *KCNT1*, *SYNGAP1*, *TUBB2A*, *MYT1L*, *NHLRC1*. A total of 59 patients (22.2%) experienced no change in their overall condition.

Supplementary table 1. Response to treatment according to gene etiology (including gene with 1 patient prescribed with CBD)		
	Seizure reduction %	CGI scale
WDR45 (1 pt)	15	3
TUBB2A (1 pt)	0	6
TUBA1A (1 pt)	0	2
TRA2B (1 pt)	0	4
TNFSF13B (1 pt)	14	
TBC1D24 (1 pt)	20	4
SZT2 (1 pt)	60	4
STAMBP (1 pt)	72	2
SPATA5 (1 pt)	10	3
SMARCA2 (1 pt)	50	1
SLC6A1 (1 pt)	0	
SLC39A8 (1 pt)	0	5
SLC2A1 (1 pt)	91	
SLC13A5 (1 pt)	75	2
SCN3A (1 pt)	80	2
SCN1B (1 pt)	30	4
SATB2 (1 pt)	85	2
RNASEH2B (1 pt)	60	
PPT1 (1 pt)	20	
PPP2CA (1 pt)	98	1
POGZ (1 pt)	100	1
PLA2G6 (1 pt)	0	4
PIGS (1 pt)	25	3
NPRL3 (1 pt)	10	3
NPC1 (1 pt)		
NF1 (1 pt)	80	
NEDD4L (1 pt)	80	2
NBEA (1 pt)	60	3
NARS2 (1 pt)	50	4
MYT1L (1 pt)	36	7
COL4A2 (1 pt)	50	2
KCNA1 (1 pt)	50	3
HECW2 (1 pt)	0	3
GRIN2B (1 pt)	10	2
GRIN1 (1 pt)	0	4
GABRG2 (1 pt)	0	4
GABRA3 (1 pt)	25	4
FBXW7 (1 pt)	0	4
EPM2A (1 pt)	80	
DHX15 (1 pt)	46	2
DHDDS (1 pt)	10	3
CNTNAP2 (1 pt)	38	3

CNKSR2 (1 pt)	60	2
CACNA1E (1 pt)	50	3
BRAF (1 pt)	60	4
ARX (1 pt)	75	1
AP1S2 (1 pt)	0	2
AHDC1 (1 pt)	52	3

**Supplementary table 2. General linear mixed model of clinical variables associated with % seizure reduction.**			
Variables	Estimated mean change	95% CI	p value
Female sex	-1.5	-12.01, 8.93	0.77
Age at seizure onset (months)	0.03	-0.13, 0.20	0.70
Follow-up duration	-0.07	-0.40, 0.27	0.70
Normal/borderline cognitive functioning (reference)			
Mild intellectual disability	-39.5	-76.87, -2.17	0.04*
Moderate/severe	-37.1	-69.34, -4.81	0.026*
Family history of epilepsy in $1^{st/2nd}$ degree relatives	-10.1	-23.10-2.80	0.13
Age at CBD prescription	0.32	-0.31, 0.95	0.32
Previously used ASM (n)	-1.4	-3.05, 0.31	0.11
Initial CBD target dose	-0.4	-1.53, 0.77	0.52
History of tonic seizures	-9.4	-20.91, 2.17	0.11
History of GPFA	5.1	-8.02, 18.21	0.45
Maximum duration of seizure freedom prior to CBD prescription (days)	0.03	0.003, 0.05	0.029*
Concomitant clobazam use	-0.4	-11.18, 10.46	0.95
Approved vs off label prescription	-6.5	-17.63, 4.56	0.25
Abbrevations: ASM = antiseizure medications; CBD = cannabidiol; GPFA = generalized paroxysmal fast activity; CI = confidence interval of beta coefficients. The asterisks indicate statistically significant variables (p<0.05)			

Supplementary Figure 1. Rate of missing data for each variable

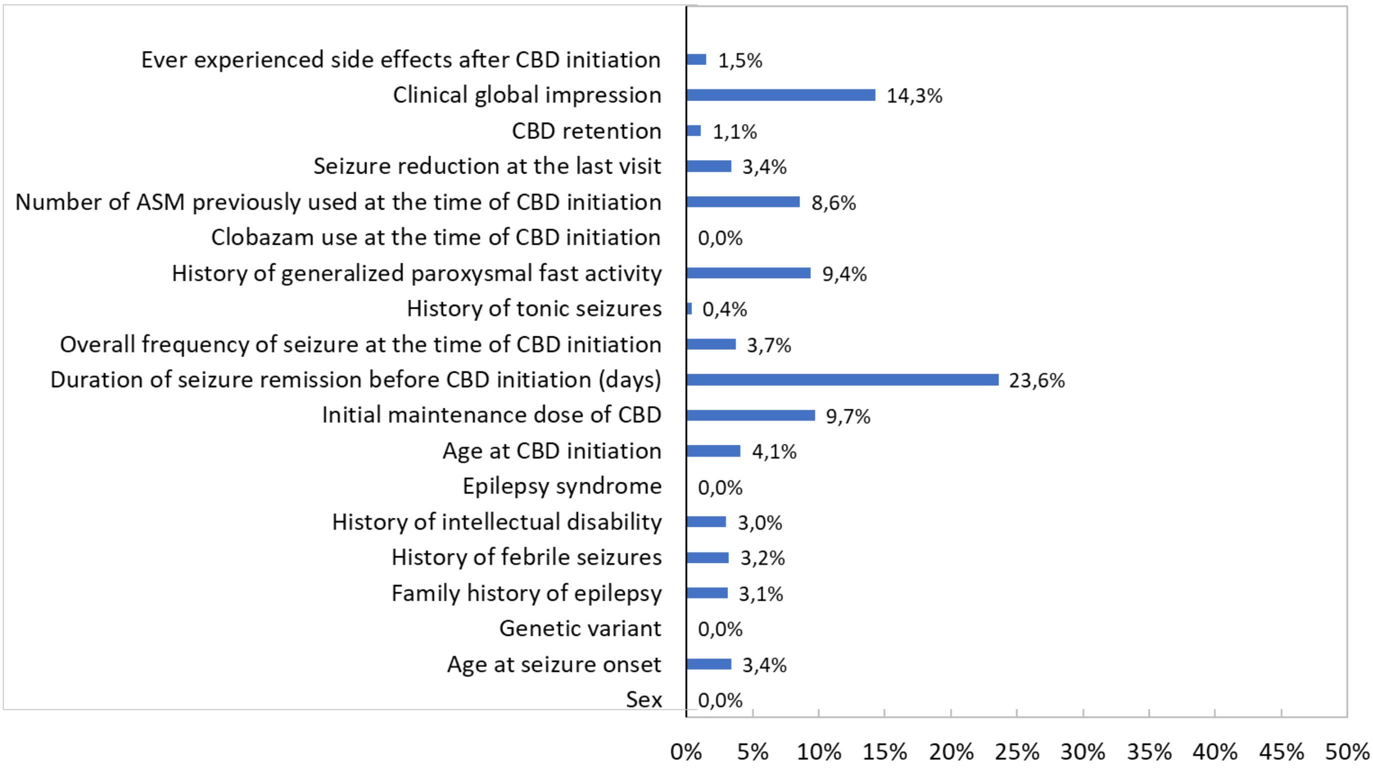

Supplementary Figure 2. Estimated mean seizure reduction according to epilepsy syndrome

Figure legend

Estimated mean seizure reduction (%) after adjustment for intellectual disability severity and maximum duration of seizure freedom prior to CBD prescription.

Estimated marginal mean for Lennox Gastaut Syndrome (LGS) was 49.8, 95% confidence interval (CI) = 33.2-66.5 ; Dravet syndrome (DS) = 52.5, 95% CI 39.2-65.8, Tuberous sclerosis complex (TSC) = 52.8, 95% CI 36.2-69.5; other developmental and epileptic encephalopathy (DEE) = 55.9, 95% CI 42.3-69.5

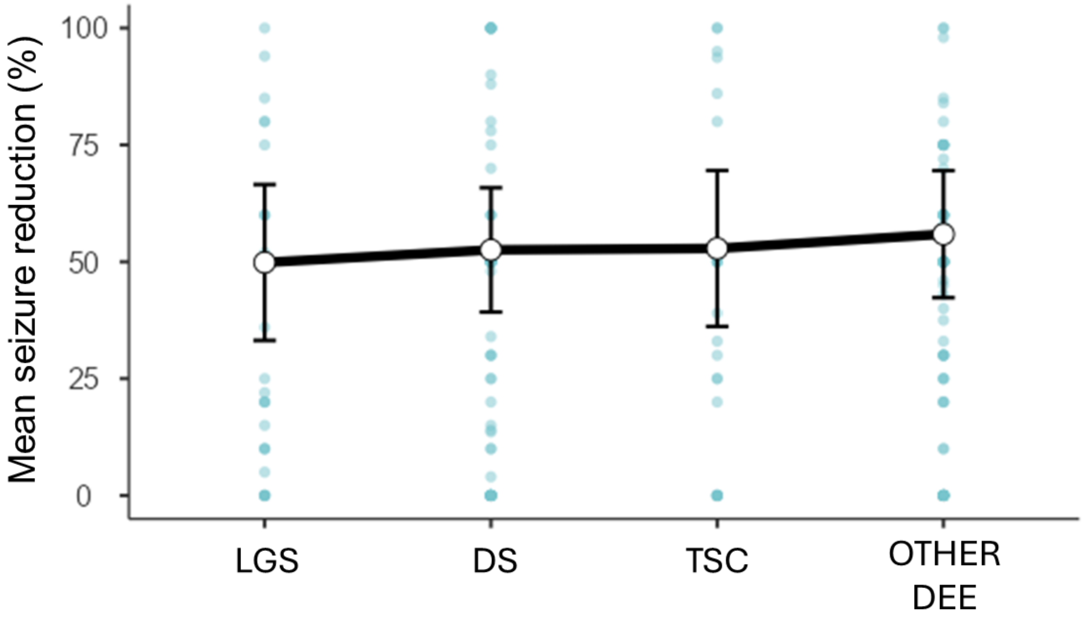

Supplement: Supplementary file 1 — Appendix S1. [file EPI-66-2253-s001.pdf]
